# Preferential pruning of inhibitory synapses by microglia contributes to alteration of the balance between excitatory and inhibitory synapses in the hippocampus in temporal lobe epilepsy

**DOI:** 10.1111/cns.14224

**Published:** 2023-04-18

**Authors:** Jianchen Fan, Xinyan Dong, Yejiao Tang, Xuehui Wang, Donghui Lin, Lifen Gong, Chen Chen, Jie Jiang, Weida Shen, Anyu Xu, Xiangnan Zhang, Yicheng Xie, Xin Huang, Linghui Zeng

**Affiliations:** ^1^ College of Pharmaceutical Sciences, Institute of Pharmacology and Toxicology, Key Laboratory of Medical Neurobiology of the Ministry of Health of China Zhejiang University Hangzhou China; ^2^ Key Laboratory of Novel Targets and Drug Study for Neural Repair of Zhejiang Province, School of Medicine Hangzhou City University Hangzhou China; ^3^ Department of Neurology The Children's Hospital, Zhejiang University School of Medicine, National Clinical Research Center For Child Health Hangzhou China; ^4^ Department of Neurosurgery The First Affiliated Hospital, Zhejiang University School of Medicine Hangzhou China

**Keywords:** E/I balance, epilepsy, microglia, synapse, synaptic phagocytosis

## Abstract

**Background:**

A consensus has formed that neural circuits in the brain underlie the pathogenesis of temporal lobe epilepsy (TLE). In particular, the synaptic excitation/inhibition balance (E/I balance) has been implicated in shifting towards elevated excitation during the development of TLE.

**Methods:**

Sprague Dawley (SD) rats were intraperitoneally subjected to kainic acid (KA) to generate a model of TLE. Next, electroencephalography (EEG) recording was applied to verify the stability and detectability of spontaneous recurrent seizures (SRS) in rats. Moreover, hippocampal slices from rats and patients with mesial temporal lobe epilepsy (mTLE) were assessed using immunofluorescence to determine the alterations of excitatory and inhibitory synapses and microglial phagocytosis.

**Results:**

We found that KA induced stable SRSs 14 days after status epilepticus (SE) onset. Furthermore, we discovered a continuous increase in excitatory synapses during epileptogenesis, where the total area of vesicular glutamate transporter 1 (vGluT1) rose considerably in the stratum radiatum (SR) of cornu ammonis 1 (CA1), the stratum lucidum (SL) of CA3, and the polymorphic layer (PML) of the dentate gyrus (DG). In contrast, inhibitory synapses decreased significantly, with the total area of glutamate decarboxylase 65 (GAD65) in the SL and PML diminishing enormously. Moreover, microglia conducted active synaptic phagocytosis after the formation of SRSs, especially in the SL and PML. Finally, microglia preferentially pruned inhibitory synapses during recurrent seizures in both rat and human hippocampal slices, which contributed to the synaptic alteration in hippocampal subregions.

**Conclusions:**

Our findings elaborately characterize the alteration of neural circuits and demonstrate the selectivity of synaptic phagocytosis mediated by microglia in TLE, which could strengthen the comprehension of the pathogenesis of TLE and inspire potential therapeutic targets for epilepsy treatment.

## Graphical abstract

In TLE circuit‐related subregions of the hippocampus, the number of excitatory synapses increased, while inhibitory synapses decreased during epileptogenesis. Microglia activated in a considerable number after epilepsy has formed, especially in stratum lucidum and polymorphic layer, which conducted phagocytosis. In TLE, microglia preferentially pruned inhibitory synapses in the hippocampus.

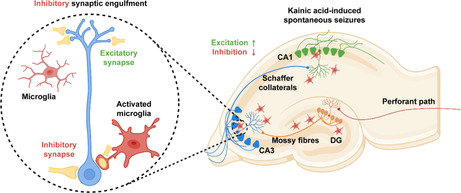

## 1 INTRODUCTION

Epilepsy is a disease characterized by recurrent seizures resulting from the hyperexcitability and hypersynchrony of neurons.^1^, ^2^ It affects over 65 million people worldwide and is triggered by a variety of factors, including traumatic brain injury, brain tumors, stroke, etc.^3^ Temporal lobe epilepsy (TLE), originating from tissue damage in the hippocampus or amygdala, is the most common form of epilepsy.^4^ Upon the onset of seizures, the brain typically undergoes reorganization of neural circuits and activities. Furthermore, people will eventually develop spatial learning and memory deficits with the progression of epilepsy.^5^ Despite the existence of effective treatments that primarily target ion channels to inhibit the abnormal firing of neurons, approximately one‐third of patients with TLE do not respond to medical treatment, making it the most prevalent form of drug refractory epilepsy.^6^ Thus, there is a critical need to elucidate the pathogenesis of epilepsy, which may facilitate the development of more effective and non‐invasive therapies.

Until now, it has been generally assumed that the emergence of epilepsy is strongly interconnected with the reconfiguration of neural circuits in the hippocampus.^7^ The trisynaptic circuit is one of the most classic neural circuits in the hippocampus in which the dendrites of granule cells in the dentate gyrus (DG) of the hippocampus receive an excitatory transmission from the perforant path axons, and then project through the mossy fibers to the dendrites of the pyramidal cells in the cornu ammonis 3 (CA3), and finally project to pyramidal cells of CA1 via Schaffer's veins.^8^, ^9^, ^10^ In this circuit, a seizure originates in a primary lesion and projects to another region where the response is intensified, even amplifying paroxysmal firing and spreading the seizure to other brain regions. The discharge of epilepsy is thus prolonged in space and time, resulting in a seizure more intense than if the primary lesion had caused seizures.^11^, ^12^

Accumulated evidence indicates that the balance between excitatory and inhibitory synapses (E/I balance) may play a crucial role in aberrant electrical signals in neural circuits.^2^, ^13^ The mainstream opinion on the underlying mechanism of epilepsy is that the E/I balance is disturbed, where excitatory synaptic transmission is enhanced while inhibitory synaptic transmission is weakened during epileptogenesis.^14^, ^15^ Glutamate is crucial for the development of mammalian learning, memory, and cognitive function. However, excessive glutamate synaptic transmission can also result in neurotoxic effects and lead to a variety of neurological disorders such as epilepsy.^16^, ^17^ The distribution and overall changes of excitatory/inhibitory synapses during epileptogenesis have been briefly studied in the hippocampus in experimental animal models and clinical brain specimens. In a rat model of TLE, it was determined that the excitatory synaptic protein vesicular glutamate transporter 1 (vGluT1) is predominantly expressed in the stratum radiatum (SR) of CA1, the stratum lucidum (SL) of CA3, the outer and inner molecular layer (OML, IML), and the polymorphic layer (PML) of the DG and subiculum (SUB) in the rat hippocampus.^18^ Moreover, vGluT1 immunoreactivity in the human epileptic hippocampus was prominent in the stratum pyramidale (SP) of the CA, SR, SL, and the IML of the dentate gyrus (DG) and SUB. Intriguingly, the intensity of vGluT1 in human epileptic hippocampal SR of CA1 and the SLM and OML of DG was significantly lower than in rats.^19^ In addition, vGluT1 protein levels were higher in all hippocampal subregions of patients with non‐hippocampal sclerosis (non‐HS) than in patients with HS and the control group.^19^, ^20^ Despite their small number, interneurons are essential for the homeostasis of neural circuits in the brain, where they attach to specific excitatory inputs and provide feedforward and feedback inhibition to pyramidal neurons, maintaining homeostasis of neural activity in the brain.^21^, ^22^ Gephyrin, an inhibitory postsynaptic protein, is widely distributed in the CA1, CA3, and DG of TLE rats.^23^ In addition, the onset of seizures reduced gephyrin expression in the hippocampus and adjacent neocortex.^24^ Furthermore, gephyrin significantly downregulated in the human temporal neocortex in patients with mesial temporal lobe epilepsy (mTLE).^25^ Although pervasive studies have documented these changes, no studies have clarified how excitatory and inhibitory synapses change in the region‐specific network of the trisynaptic circuit during epileptogenesis. Moreover, the expression of glutamate decarboxylase 65 (GAD65) was found to increase in Ihara's epileptic rats and older spontaneously epileptic rats.^26^ GAD65 in remaining hippocampal GABA neurons also increased in pilocarpine‐treated rats.^27^ However, a decrease of GAD65 was observed in the sensorimotor cortex in TLE.^28^ Given these, controversy still exists regarding the alteration of the number of synapses in different epilepsy nidus and models.

Glia cells are vital components of nerve cells, with microglia and astrocytes constituting the largest population.^29^, ^30^, ^31^ Abnormal activation and release of inflammatory mediators by microglia and astrocytes exacerbate epileptogenesis.^32^, ^33^, ^34^ However, recent evidence has pointed out that microglia and astrocytes also contain anticonvulsant properties.^35^, ^36^, ^37^ Microglia are resident immune cells in the brain^38^ that engulf cell debris, facilitate neural development, and prune synapses, thereby preserving brain homeostasis.^31^ In the physiological microenvironment of the brain, microglia assume a ramified form to maintain the structure and function of synapses via their processes.^39^ Diseases such as epilepsy lead to the formation of ameboid microglia, which prune abnormally formed synapses.^39^, ^40^, ^41^, ^42^ In some cases, however, microglia can also prune both excitatory and inhibitory synapses, but the E/I ratio continues to favor excitation.^43^, ^44^ Interestingly, microglia tend to prune synapses with less active inputs.^45^ Unfortunately, the selectivity of microglial phagocytosis in epilepsy is unknown. Furthermore, it remains to be determined how the E/I balance shifts in epilepsy after microglial pruning.

In this study, we generated a kainic acid (KA)‐induced epilepsy model in rats, which were examined at various time points to explore the changes in the number of excitatory and inhibitory synapses, along with the preference for microglial phagocytosis in the hippocampus. Electroencephalography (EEG) recording demonstrated stable spontaneous recurrent seizures (SRS) in rats 14 days after KA administration. We observed a continuous increase in excitatory synapses during epileptogenesis, while inhibitory synapses decreased significantly. In addition, vGluT1 occupied a high proportion in the SR, SL and PML during epileptogenesis. Moreover, we found that microglia preferentially pruned inhibitory synapses after the formation of recurrent seizures. Consistent results were also validated regarding the selectivity of microglial phagocytosis in the resected hippocampus in mTLE patients. Taken together, these findings can aid in elucidating the potential mechanism by which microglia contribute to synaptic alterations in TLE‐derived neural circuits, inspiring novel therapeutic interventions targeting microglia for TLE along with its complications.

## 2 MATERIALS AND METHODS

### 2.1 Animals

Male Sprague Dawley (SD) rats (180–200 g) were used and purchased from Shanghai SLAC Laboratory Animal Co., Ltd (certificate: SCXK 2022–0004). Animals were kept in the Laboratory Animal Center of Zhejiang University and were group housed in three per cage, in stainless cages under a 12‐h/12‐h light/dark cycle at room temperature of 22 ± 1°C, humidity of 50 ± 20% with food and water ad libitum. The animal protocols were approved by the Animal Care and Use Committees at the Zhejiang University School of Medicine and were conducted in accordance with the policies of institutional guidelines on the care and use of laboratory animals.

### 2.2 Human samples

The human cases investigated in this study were obtained from the First Affiliated Hospital, Zhejiang University School of Medicine. The information of patients is shown in Table 1. One brain slice of every patient was used for Nissl staining to confirm that the site of surgery was taken in the temporal lobe and hippocampus. Otherwise, the patients were excluded. Patients (*n* = 6) underwent surgical resection treatment of a varying part of the temporal lobe for medically intractable TLE. The whole protocols were approved by the Ethics Committee of the First Affiliated Hospital, Zhejiang University School of Medicine (No. 531) and followed the guidelines of the Declaration of Helsinki. Informed consent was obtained from the families concerned before specimen collection.

**TABLE 1:** Clinical data of the patients.

ID	Diagnosis	Sex	Age at surgery (years)	Epilepsy duration (years)	Site of surgery
1	TLE	Male	24	12	TH
2	TLE	Female	45	40	TH
3	TLE	Female	25	10	H
4	TLE	Female	18	15	H
5	TLE	Male	38	30	TH
6	TLE	Male	26	13	TH

Abbreviations: H, hippocampus; T, temporal lobe.

### 2.3 Kainic acid‐induced epilepsy model

Rats were randomly assigned to one of five groups: control group, which received an intraperitoneal (i.p.) injection of saline, and 7, 14, 28, and 63 days following the induction of epileptic seizure, which received an i.p. injection of 18 mg/kg kainic acid (ab120100, Abcam). Each group consisted of 6 SD rats. The Racine scores were applied to rate seizure severity: category 1, immobility and facial twitch; category 2, head nodding; category 3, forelimb clonus; category 4, rearing; and category 5, rearing and falling.^46^ According to previous studies, the onset of epilepsy was defined when at least two generalized motor seizures (stage 4–5) were observed 48 h post status epilepticus.^47^, ^48^ Seizures were terminated by 0.1% diazepam (10 mg/kg) when the rats exhibited status epilepticus, 3 h after the KA induction. An additional injection was given at 50% of the initial dose every 1 h after the KA injection if the rats did not exhibit category 4–5 seizures to make them develop category 4–5 seizures. Animals did not show stage 4 seizures after two additional applications of KA would not be involved in the subsequent study. Among the rats that exhibited stage 4–5 seizures, six rats were randomly drawn for EEG recording to verify that the dose of KA led to stable SRSs. Among the remaining rats, we screened out those exhibiting stage 4–5 seizures 14 days after KA injection for subsequent studies.^47^, ^48^

### 2.4 Tissue preparation

The human cases were fixed in 10% formalin fixing solution (P2161, Solarbio, CHN) and dehydrated with ethanol. After paraffin embedding, 5 μm sections were cut using a rotary slicer (HM325, Thermo) and were prepared for Nissl staining and immunofluorescence. Anesthetized rats were transcardially perfused with phosphate‐buffered saline (PBS), after which the rat brains were fixed with 4% paraformaldehyde (PFA) overnight at 4°C, then moved to a 15% sucrose solution overnight, and finally to a 30% sucrose solution. A sliding microtome (HM520, Microm) was used to cut 30 μm brain slices, which were then preserved in a cryoprotectant at −20°C. Three animals per group were used to harvest 3–4 matching slices between 4.56‐ and 3.60‐mm posterior to the bregma in one hemisphere of the rat brain for each experiment.

### 2.5 Nissl staining

For Nissl staining, 5 μm thick sections were dewaxed to distilled water and stained with Cresyl Violet Stain (G1430, Solarbio, CHN) in 56°C incubator for 1 h. Then the sections were differentiated by Nissl Differentiation Solution (G1430, Solarbio, CHN) for 20 s, and were subsequently dehydrated in absolute ethanol, transparented by xylene and finally sealed with resinene.

### 2.6 Immunofluorescence and imaging

For immunofluorescence, brain sections were blocked for 1 h with QuickBlockTM blocking buffer for Immunol labeling (P0260, Beyotime, CHN) and then incubated overnight at 4°C with primary antibody diluted in the blocking solution (rabbit anti‐IBA1, 1:500, PTR2404; mouse anti‐CD68, 1:100, ab955, Abcam^49^; guinea pig anti‐vGluT1, 1:1000, ab5905, Millipore, mouse anti‐GAD65, 1:50, ab_528264, Developmental Studies Hybridoma Bank). After washing, the sections were incubated with corresponding secondary antibodies: Alexa 488‐conjugated goat anti‐mouse IgG (1:1000, A21121, Invitrogen), Alexa 488‐conjugated goat anti‐guinea pig IgG (1:1000, A11073, Invitrogen), Alexa 568‐conjugated goat anti‐mouse IgG (1:1000, A21134, Invitrogen) or Alexa 647‐conjugated goat anti‐rabbit IgG (1:1000, A32733, Invitrogen) for 2 h at 37°C temperature; then slices were mounted on glass coverslips with DAPI Fluoromount‐G (0100–20, Southern Biotech). The sections were imaged by a laser scanning spectral confocal microscope (TCS SP8, Leica Microsystems).

### 2.7 Synaptic puncta staining and analysis

All confocal images of vGluT1 or GAD65 were obtained using Z‐stack (1 μm step) with a 63‐objective oil with 2× zoom. Synaptic puncta analysis was performed with ImageJ. For all rats in the experiment, images were captured with a 1 airy unit (AU) pinhole while keeping the gain and offset settings constant. Three sites per slice were captured for analyzing. For the analysis, the Gaussian blur of each image was set to 1 and all contrasted photos were set to the same threshold, then the total area of vGluT1 and GAD65 were calculated in the same regions of each rat, Age‐matched control rats were employed to exclude the age‐related dynamics of all parameters, the results of rats at different ages were normalized to the age‐matched control (Figure S1, S2). For display, post‐analysis photos were brightened and contrasted over the whole image.

The lysosomal marker, immunolabeling of CD68 was applied to identify the phagocytic microglia. Co‐labeling of lysosomal marker CD68 and vGluT1 or GAD65 was used to evaluate the synaptic pruning of microglia. Sections were imaged with the 63× objective oil with 2× zoom using the confocal microscope at 1 μm Z‐stack step. Images on each stack were overlaid for microglia counting. Three sites per slice were selected for counting. ImageJ was used to remove the noise from the image. The Gaussian blur per image was set to 1, and plot profile in ImageJ was used to reconstruct the microglia and synaptic volumes. The number of IBA1 positive cells co‐stained with DAPI was counted manually.

### 2.8 Electroencephalography recording

The KA model of epilepsy has been expounded previously. Rats typically develop SRSs 2 weeks after the onset of status epilepticus (SE).^50^ 14 days after KA injection, rats were surgically implanted with epidural electrodes in the cortex for EEG recording. The rats were first anesthetized with 3% pentobarbital sodium. Then recording electrodes were implanted on the left side of the rat (AP: − 4.08 mm; ML: 3.6 mm; DV: − 0.8 mm). And two screws were placed on the right and inferior sides of the cerebellum's skull to serve as reference and ground electrodes for EEG recording. The screws were welded through copper wire and fixed to the electronic pins with dental cement. Rats were allowed 7 days of recovery following the surgery. Rats were placed in a 35 cm^2^ square cage to move freely for 1 day prior to EEG recording at a sampling rate of 1 kHz (Spike 2, CED) synchronized with a digital video monitoring system. EEG was acquired using standard alternating current amplifiers without bandpass filters. Rats were monitored 12 h per day for seven consecutive days. EEG data analysis was carried out by two trained observers who were not aware of the experimental groups. The video was evaluated when necessary to rule out sources of artifacts. All EEG data from each monitoring session was reviewed for hippocampal paroxysmal discharges (HPDs). The baseline of epilepsy in each rat is stable and reproducible, and seizures do not occur in clusters, which is a key prerequisite for reliable pharmacological studies. HPDs were defined as a spike in frequency ≥ 2 Hz, amplitudes at least 3‐fold higher than baseline and duration longer than 10 s. Spikes with a baseline of fewer than 5 s between them were deemed to be the same seizure.^51^ The number of HPDs, seizure duration and spiking amplitude per 24 h were counted.

### 2.9 Statistical analysis

GraphPad Prism 8.0 software (GraphPad Software Inc.) was used for graphing and analysis. Data that do not exhibit a Gaussian distribution were analyzed via a non‐parametric equivalent. Unpaired student's *t* test was used for two groups. One‐way ANOVA with Tukey's post hoc test or Kruskal‐Wallis with Dunn's multiple comparisons test (nonparametric) was used for multiple groups. Correlations were calculated using the number of CD68^+^ microglia and the number of activated microglia. Equation was made by liner regression analyzes. Correlation analyzes was performed by computing Pearson's correlation coefficient. Data in this study are presented as mean ± standard error (SEM). Each experiment was performed with at least three biological replicates, and *p* < 0.05 was considered statistically significant.

## 3 RESULTS

### 3.1 Kainic acid induces spontaneous recurrent seizures 14 days after status epilepticus onset

**FIGURE 1:**
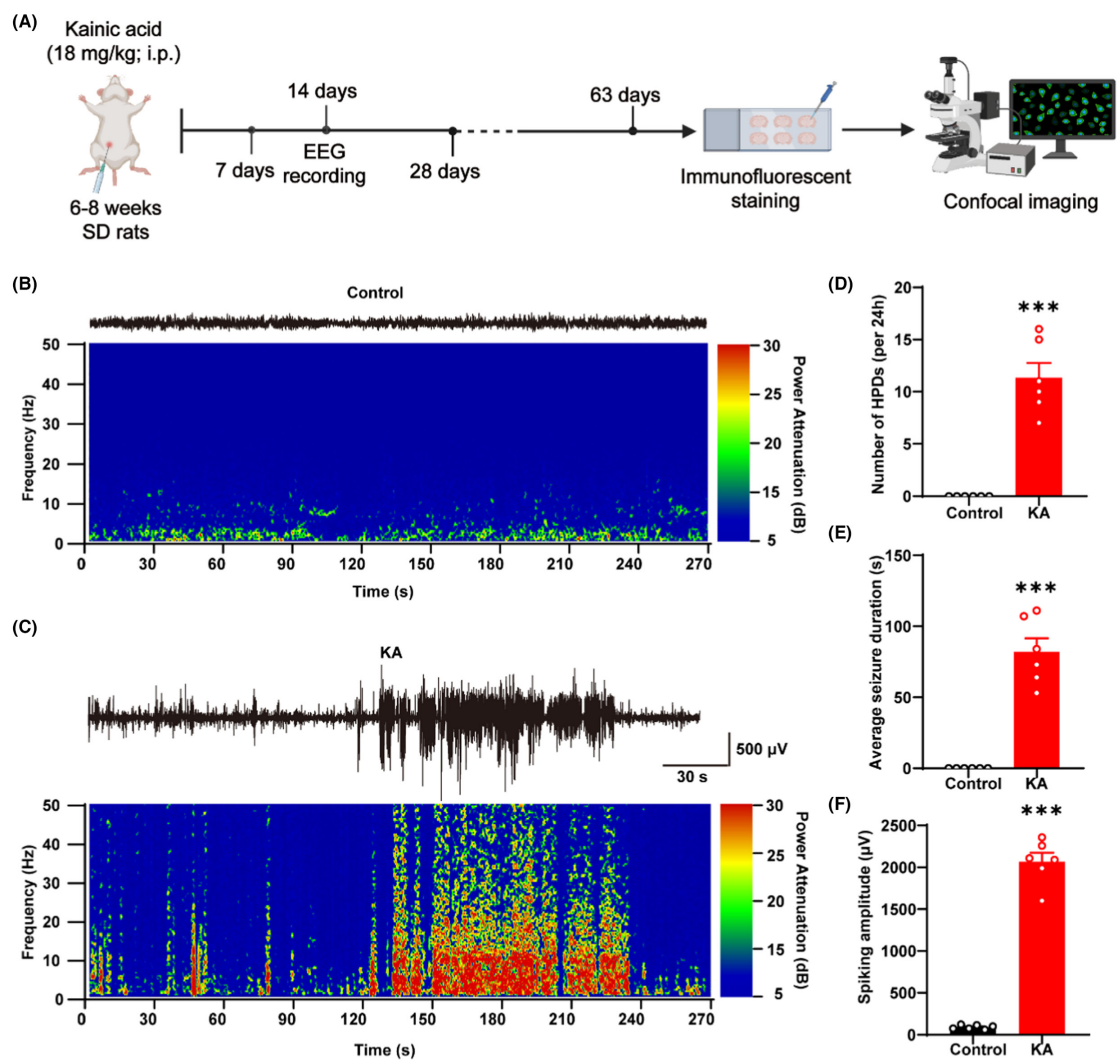
Kainic acid induces stable spontaneous recurrent seizures in rats. (A) The schematic diagram of experimental design. (B, C) Representative EEG recordings and energy spectra of the control and KA‐induced rats. (D–F) Quantification of the number of HPDs, average seizure duration and spiking amplitude indicates the SRSs in rats 14 days after KA induction. *: vs. control, *p < 0.05, **p < 0.01, ***p < 0.001; unpaired student's *t* test. Data are expressed as the mean ± SEM. *n* = 6 rats per group.

Previous studies demonstrated that the number of synapses could be altered in TLE.^52^ Here, we conducted comprehensive research on the number of synapses in the hippocampus of rats, which was detailed at various time points following KA‐induced TLE and in various hippocampal subregions. We first established a KA‐induced TLE model (i.p. injection of 18 mg/kg) in SD rats. The brains of TLE rats were sectioned at 7, 14, 28 and 63 days following KA induction for immunofluorescent staining and confocal microscopy image analysis (Figure 1A). Since stable and reproducible baseline of epilepsy is a key prerequisite for reliable pharmacological studies, EEG recording was operated 14 days after KA induction to validate whether rats developed stable SRSs. The EEG results showed that KA induced significant elevation of the HPDs, seizure duration and spiking amplitude 14 days after SE onset compared with the control rats (number of HPDs: ***p < 0.001, 11.33 ± 1.43 vs. 0.00 ± 0.00, KA vs. control group, seizure duration: ***p < 0.001, 82.00 ± 9.52 vs. 0.00 ± 0.00, KA vs. control group, spiking amplitude: ***p < 0.001, 2068.00 ± 107.90 vs. 92.33 ± 10.28, KA vs. control group, Figure 1D–F). Furthermore, the EEGs and corresponding energy spectra of seizure activity clearly demonstrated the epileptiform spikes in KA‐induced rats (Figure 1B,C).

### 3.2 Kainic acid induces the alteration of excitatory synapses in rats' hippocampus

**FIGURE 2:**
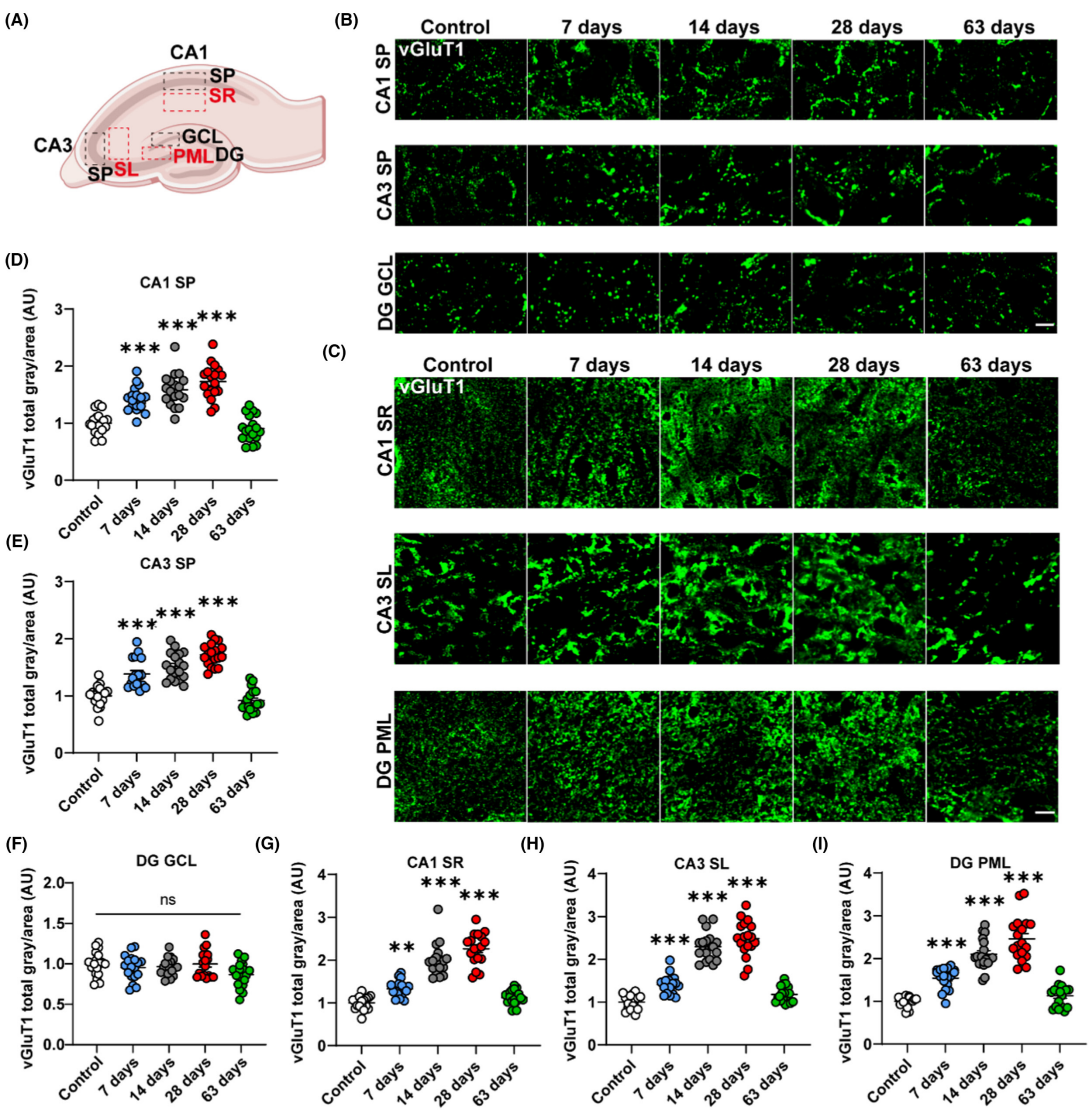
Kainic acid induces the alteration of excitatory synapses in the rats' hippocampus at different time points. (A) The regions of interest are shown in the hippocampus in the schematic diagram. (B, C) Representative vGluT1 (green) immunostaining from the functional regions of the CA1, CA3 and DG 7, 14, 28, and 63 days after KA induction. Scale bar = 10 μm. (D, E) Quantification of the total area of vGluT1 presents a gradual upwards tendency in the SP of CA1 and CA3 from days 7 to 28, which decreased to the control levels on day 63. (F) Quantification of the total area of vGluT1 reveals no obvious changes in the GCL of the DG at various time points following KA induction. (G–I) Quantification of the total area of vGluT1 presents a gradual upwards tendency in the SR of the hippocampal CA1, SL of CA3 and PML of DG from days 7 to 28, which decreases to the control levels on day 63. *: vs. control, *p < 0.05, **p < 0.01, ***p < 0.001, one‐way ANOVA with Tukey's post hoc test. Data were normalized to the control group. Data are expressed as the mean ± SEM. *n* = 18 images from six rats per group with three brain sections per rat.

To explore the changes in excitatory synapses, we evaluated the changes in vGluT1, an excitatory synapse marker, in each functional region of the hippocampal CA1, CA3, and DG after KA induction (Figure 2A–C). The quantitative analysis of immunofluorescence in rats' hippocampus indicated that in the SP of CA1 and CA3, KA induced a significant elevation of the vGluT1^+^ area during periods of epileptogenesis (7–28 days) and returned to control levels in a chronic phase of epilepsy (63 days) (CA1 SP: ***p < 0.001, 1.42 ± 0.05 vs. 1.00 ± 0.04, 7 days vs. control group, ***p < 0.001, 1.59 ± 0.07 vs. 1.00 ± 0.04, 14 days vs. control group, ***p < 0.001, 1.73 ± 0.07 vs. 1.00 ± 0.04, 28 days vs. control group; CA3 SP: ***p < 0.001, 1.38 ± 0.06 vs. 1.00 ± 0.04, 7 days vs. control group, ***p < 0.001, 1.51 ± 0.06 vs. 1.00 ± 0.04, 14 days vs. control group, ***p < 0.001, 1.72 ± 0.05 vs. 4.94 ± 0.23, 28 days vs. control group, Figure 2D,E). In the granular cell layer (GCL) of the DG, however, there were no discernible changes in the vGluT1^+^ area across these time points following KA induction (Figure 2F). Regarding the SR of the hippocampal CA1, the SL of CA3 and the PML of DG, the vGluT1^+^ area elevated incrementally from day 7 to 28, but returned to the control levels on day 63 (SR: **p < 0.01, 1.33 ± 0.04 vs. 1.00 ± 0.04, 7 days vs. control group, ***p < 0.001, 2.00 ± 0.09 vs. 1.00 ± 0.04, 14 days vs. control group, ***p < 0.001, 2.26 ± 0.09 vs. 1.00 ± 0.04, 28 days vs. control group; SL: ***p < 0.001, 1.41 ± 0.05 vs. 1.00 ± 0.04, 7 days vs. control group, ***p < 0.001, 2.29 ± 0.08 vs. 1.00 ± 0.04, 14 days vs. control group, ***p < 0.001, 2.49 ± 0.10 vs. 1.00 ± 0.04, 28 days vs. control group; PML: ***p < 0.001, 1.54 ± 0.06 vs. 1.00 ± 0.03, 7 days vs. control group, ***p < 0.001, 2.10 ± 0.08 vs. 1.00 ± 0.03, 14 days vs. control group, ***p < 0.001, 2.46 ± 0.12 vs. 1.00 ± 0.03, 28 days vs. control group, Figure 2G–I). Together, the general elevation of excitatory synapses suggests an increased excitability in the hippocampus in the KA‐induced rat model of TLE from days 7 to 28, while the decreases in excitatory synapses on day 63 imply cognitive impairment‐related synaptic loss.

### 3.3 Kainic acid induces the alteration of inhibitory synapses in rats' hippocampus

**FIGURE 3:**
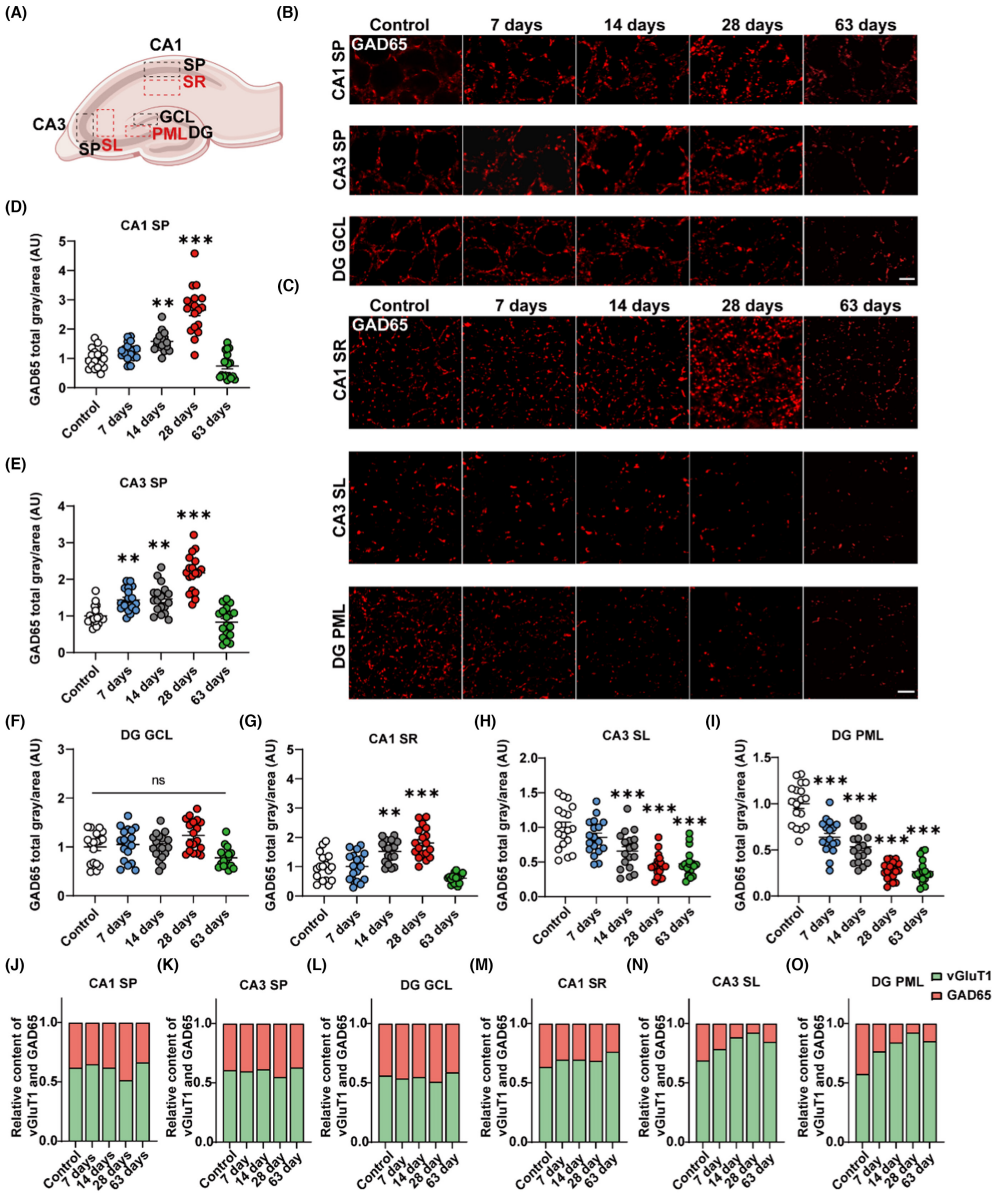
Kainic acid induces the alteration of inhibitory synapses in the rats' hippocampus at different time points. (A) The regions of interest are shown in the hippocampus in the schematic diagram. (B, C) Representative GAD65 (red) immunostaining from the functional regions of the CA1, CA3 and DG 7, 14, 28, and 63 days after KA induction. Scale bar = 10 μm. (D, E) Quantification of the total area of GAD65 presents a gradual augmentation in SP of the CA1 and CA3 from days 7 to 28 and decreases to the control levels on day 63 after KA induction. (F) The total area of GAD65 shows no obvious changes in the GCL of DG at various time points following KA induction. (G) Quantification of the total area of GAD65 shows a gradual upwards tendency in the SR of the hippocampal CA1 from days 7 to 28, and decreases to the control levels on day 63 after KA induction. (H, I) Quantification of the total area of GAD65 shows a reduction in the SL of the CA3 and PML of the DG from days 7 to 63 after KA induction. (J–O) The relative content of vGluT1 and GAD65 shows a higher percentage of excitatory synapses compared to the control group in the SR, SL and PML but an inconspicuous propensity in the SP and GCL. *: vs. control, *p < 0.05, **p < 0.01, ***p < 0.001; one‐way ANOVA with Tukey's post hoc test. Data were normalized to the control group. Data are expressed as the mean ± SEM. *n* = 18 images from six rats per group with three brain sections per rat.

To quantify the changes in inhibitory synapses, we used immunostaining for GAD65, an inhibitory synapse marker in each functional region of the hippocampal CA1, CA3, and DG after KA induction (Figure 3A–C). The quantitative analysis of immunofluorescence in the rat hippocampus indicated that KA induced a distinct increase in the GAD65^+^ area in the SP of CA1 on days 14 and 28. The GAD65^+^ area in the SP of the CA3 increased significantly during epileptogenesis (days 7–28), which may signal the aggregation of inhibitory synapses, and returned to baseline levels during the chronic phase of epilepsy (day 63) (CA1 SP: **p < 0.01, 1.58 ± 0.08 vs. 1.00 ± 0.08, 14 days vs. control group, ***p < 0.001, 2.65 ± 0.19 vs. 1.00 ± 0.08, 28 days vs. control group; CA3 SP: **p < 0.01, 1.44 ± 0.07 vs. 1.00 ± 0.06, 7 days vs. control group, **p < 0.01, 1.46 ± 0.09 vs. 1.00 ± 0.06, 14 days vs. control group, ***p < 0.001, 2.17 ± 0.12 vs. 1.00 ± 0.06, 28 days vs. control group, Figure 3D,E). In the GCL of the DG, however, no changes in the GAD65^+^ area were noted across these time points following KA induction (Figure 3F). Concerning the SR of the hippocampus, the GAD65^+^ area increased significantly in CA1 on days 14 and 28, and dramatically dropped during a chronic phase of epilepsy (SR: **p < 0.01, 1.52 ± 0.09 vs. 1.00 ± 0.11, 14 days vs. control group, ***p < 0.001, 1.81 ± 0.12 vs. 1.00 ± 0.11, 28 days vs. control group, Figure 3G). The total area of GAD65 decreased remarkably relative to the control and exhibited a gradual downward trend from days 14 to 63 in the SL, and from days 7 to 63 in the PML (SL: ***p < 0.001, 0.66 ± 0.06 vs. 1.00 ± 0.07, 14 days vs. control group, ***p < 0.001, 0.45 ± 0.04 vs. 1.00 ± 0.07, 28 days vs. control group, ***p < 0.001, 0.47 ± 0.05 vs. 1.00 ± 0.07, 63 days vs. control group; PML: ***p < 0.001, 0.64 ± 0.04 vs. 1.00 ± 0.05, 7 days vs. control group, ***p < 0.001, 0.53 ± 0.04 vs. 1.00 ± 0.05, 14 days vs. control group, ***p < 0.001, 0.27 ± 0.02 vs. 1.00 ± 0.05, 28 days vs. control group, ***p < 0.001, 0.26 ± 0.03 vs. 1.00 ± 0.05, 63 days vs. control group, Figure 3H,I). Furthermore, the total area of vGluT1 occupied a sizable proportion in the SR, SL and PML from day 7 to 63 and presented an increasing trend from day 7 to 28 in the SL and PML (Figure 3M–O). These findings suggest reduced inhibition in the hippocampus in the KA‐induced rat model of TLE, which denotes a shift of the E/I balance towards excitation.

### 3.4 Kainic acid‐induced activated microglia conduct phagocytosis in the rats' hippocampus during spontaneous recurrent seizures

**FIGURE 4:**
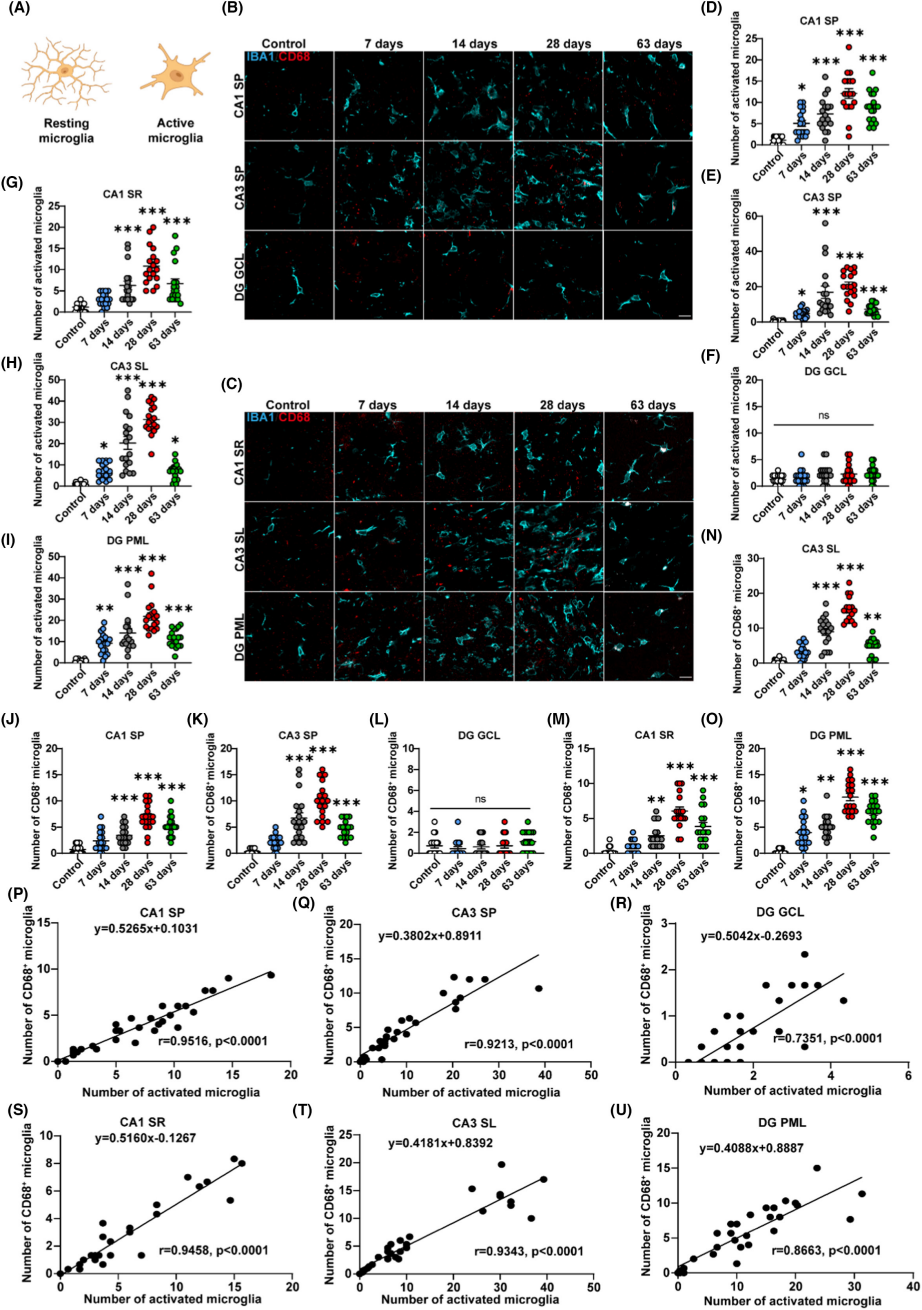
Kainic acid‐induced activated microglia conduct phagocytosis in the rats' hippocampus during the phase of recurrent seizures. (A) The schematic diagram shows the resting and activated states of microglia. (B, C) Representative confocal images display IBA1^+^ microglia in the functional regions of the CA1, CA3, and DG of the hippocampus at 7, 14, 28, and 63 days after KA induction. Scale bar = 20 μm. (D–I) Quantification of the numbers of activated microglia indicates a significant rise in the CA1, CA3, and DG of the hippocampus from days 7 to 63 after KA induction compared to the control rats, except the GCL of the DG, which demonstrates no obvious changes. (J–O) Quantification of the number of CD68^+^ microglia illustrates a significant increase in the CA1, CA3, and DG of the hippocampus from days 14 to 63 after KA induction compared to control rats, except the GCL of the DG, which exhibit no obvious changes. (P–U) Correlation analysis between activated microglia and CD68^+^ microglia in the SR, SL, and PML is depicted. The equation is calculated via linear regression analyses. Correlation analyses are performed by computing Pearson's correlation coefficient. *p < 0.05; **p < 0.01; ***p < 0.001; (H, J) one‐way ANOVA with Tukey's post hoc test; D‐G, I, K‐O: Kruskal‐Wallis with Dunn's multiple comparisons test. Data are expressed as the mean ± SEM. The quantification is from a one‐time experiment with three samples per group. Scale bar = 20 μm.

Microglia are the primary immune cells in the central nervous system. In the physiological state, ramified microglia scan the brain's environment with their processes, remodeling synapses and conducting phagocytosis. Under pathological conditions, microglia adopt an amoeboid shape, as observed in both a mouse model of KA‐induced status epilepticus and human patients with mTLE.^39^, ^53^ To explore the function of microglia in synapse pruning after TLE, immunofluorescence staining was performed for IBA1, a microglial marker gene, and CD68, a marker of lysosomes, to examine the activation and phagocytosis of microglia in the CA1, CA3, and DG of the hippocampus following KA induction. The phagocytic microglia were analyzed for the presence of microglia (IBA1^+^) that acquire a lysosome identity (CD68^+^) (Figure 4B,C). The schematic diagram depicts two morphologies of microglia (Figure 4A). Resting microglia possess ramified branches, while activated microglia show reduced process numbers and lengths with enlarged cell bodies. The quantitative analysis of immunostaining in the SP of the CA1 area revealed a large number of activated microglia, with many demonstrating phagocytic function from days 14 to 63 (activated microglia: *p < 0.05, 5.06 ± 0.72 vs. 1.11 ± 0.21, 7 days vs. control group, ***p < 0.001, 7.28 ± 0.91 vs. 1.11 ± 0.21, 14 days vs. control group, ***p < 0.001, 12.11 ± 1.15 vs. 1.11 ± 0.21, 28 days vs. control group, ***p < 0.001, 8.83 ± 0.84 vs. 1.11 ± 0.21, 63 days vs. control group; CD68^+^ microglia: ***p < 0.001, 3.44 ± 0.41 vs. 0.72 ± 0.19, 14 days vs. control group, ***p < 0.001, 7.00 ± 0.59 vs. 0.72 ± 0.19, 28 days vs. control group, ***p < 0.001, 5.22 ± 0.46 vs. 0.72 ± 0.19, 63 days vs. control group, Figure 4D,J). On days 14, 28 and 63, a remarkable augmentation of the number of activated and CD68^+^ microglia was observed in the SP of the CA3 compared to the control group (activated microglia: *p < 0.05, 4.89 ± 0.52 vs. 0.67 ± 0.14, 7 days vs. control group, ***p < 0.001, 16.89 ± 3.47 vs. 0.67 ± 0.14, 14 days vs. control group, ***p < 0.001, 20.89 ± 1.77 vs. 0.67 ± 0.14, 28 days vs. control group, ***p < 0.001, 7.28 ± 0.70 vs. 0.67 ± 0.14, 63 days vs. control group, CD68^+^ microglia: ***p < 0.001, 6.72 ± 1.02 vs. 0.33 ± 0.11, 14 days vs. control group, ***p < 0.001, 10.00 ± 0.80 vs. 0.33 ± 0.11, 28 days vs. control group, ***p < 0.001, 4.67 ± 0.39 vs. 0.33 ± 0.11, 63 days vs. control group, Figure 4E,K). Consistent with the changes in the number of synapses, the number of activated and phagocytic microglia in the SP of the DG did not vary significantly (Figure 4F,L).

In addition, the activation and phagocytosis of microglia in the SR, SL and PML were investigated. In the SR of CA1, we perceived abundant activated microglia on from days 14 to 63, whereas in the SL of CA3 and PML of the DG, activated microglia were discerned from days 7 to 63 (SR: ***p < 0.001, 6.28 ± 1.01 vs. 1.17 ± 0.23, 14 days vs. control group, ***p < 0.001, 10.83 ± 1.04 vs. 1.17 ± 0.23, 28 days vs. control group, ***p < 0.001, 6.72 ± 1.12 vs. 1.17 ± 0.23, 63 days vs. control group; SL: *p < 0.05, 7.22 ± 0.84 vs. 0.89 ± 0.20, 7 days vs. control group, ***p < 0.001, 20.28 ± 2.99 vs. 0.89 ± 0.20, 14 days vs. control group, ***p < 0.001, 31.39 ± 1.63 vs. 0.89 ± 0.20, 28 days vs. control group, *p < 0.05, 7.11 ± 0.84 vs. 0.89 ± 0.20, 63 days vs. control group; PML: **p < 0.01, 9.22 ± 1.13 vs. 0.83 ± 0.19, 7 days vs. control group, ***p < 0.001, 14.06 ± 2.06 vs. 0.83 ± 0.19, 14 days vs. control group, ***p < 0.001, 21.83 ± 1.75 vs. 0.83 ± 0.19, 28 days vs. control group, ***p < 0.001, 11.56 ± 0.98 vs. 0.83 ± 0.19, 63 days vs. control group, Figure 4G–I). Nonetheless, a striking prevalence of phagocytic microglia existed in the SR and SL from days 14 to 63 (SR: **p < 0.01, 2.50 ± 0.39 vs. 0.28 ± 0.14, 14 days vs. control group, ***p < 0.001, 6.06 ± 0.58 vs. 0.28 ± 0.14, 28 days vs. control group, ***p < 0.001, 3.83 ± 0.58 vs. 0.28 ± 0.14, 63 days vs. control group; SL: ***p < 0.001, 8.94 ± 1.00 vs. 0.33 ± 0.14, 14 days vs. control group, ***p < 0.001, 15.28 ± 0.80 vs. 0.33 ± 0.14, 28 days vs. control group, **p < 0.01, 4.89 ± 0.53 vs. 0.33 ± 0.14, 63 days vs. control group, Figure 4M,N). From days 7 to 63 after KA induction, abundant CD68^+^ microglia were also detected in the PML (*p < 0.05, 3.94 ± 0.64 vs. 0.28 ± 0.11, 7 days vs. control group, **p < 0.01, 5.17 ± 0.49 vs. 0.28 ± 0.11, 14 days vs. control group, ***p < 0.001, 10.72 ± 0.69 vs. 0.28 ± 0.11, 28 days vs. control group, ***p < 0.001, 7.78 ± 0.51 vs. 0.28 ± 0.11, 63 days vs. control group, Figure 4O). In addition, we discovered a linear correlation between the number of activated microglia and CD68^+^ microglia in the CA1, CA3 and DG regions of the hippocampus (Figure 4P–U). These results confirmed that activated microglia conducted phagocytosis during the period of SRS in the KA‐induced TLE models.

### 3.5 Kainic acid‐induced activated microglia preferentially prune inhibitory synapses in the rats' hippocampus during spontaneous recurrent seizures

**FIGURE 5:**
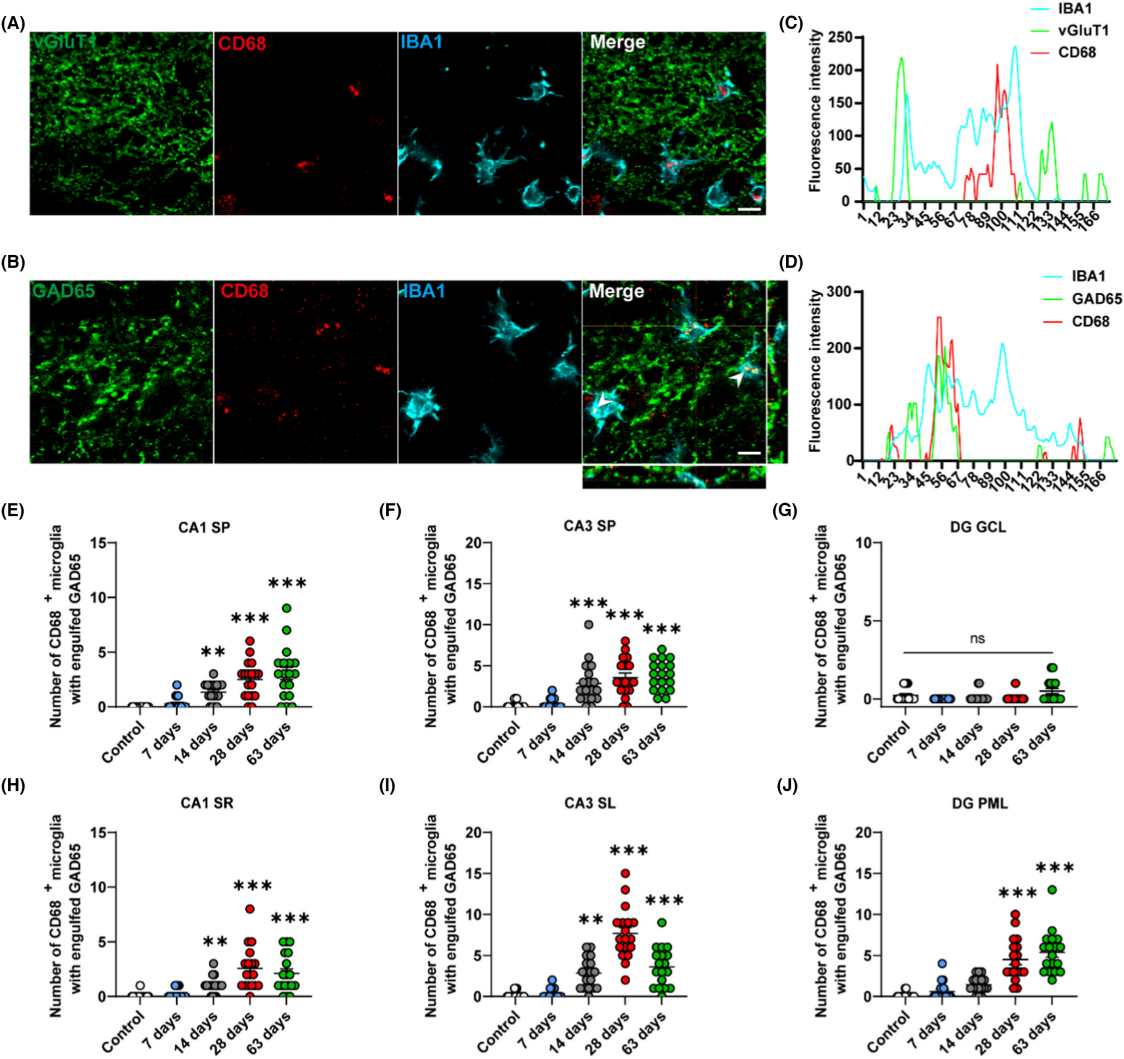
Kainic acid‐induced activated microglia preferentially prunes inhibitory synapses in the rats' hippocampus. (A) Representative confocal images are shown for CD68 (red), vGluT1 (green), and IBA1 (cyan) immunoreactive puncta in the hippocampal CA1 63 days after KA induction. (B) Immunostaining results are presented for CD68 (red), GAD65 (green) and IBA1 (cyan) in the hippocampal CA1 63 days after KA induction. High magnification confocal images display colocalization of the CD68 and GAD65 signals. (C) Quantification of the CD68 and vGluT1 immunofluorescent signals within IBA1^+^ microglia denotes no colocalization of lysosomes and excitatory synapses during KA‐induced recurrent seizures. (D) Quantification of CD68 and GAD65 immunofluorescent signals within IBA1^+^ microglia indicates phagocytosis of inhibitory synapses by microglia lysosomes during KA‐induced recurrent seizures. (E–J) Quantification of the number of CD68^+^ microglia with engulfed GAD65 illustrates a significant increase in the CA1 and CA3 of the hippocampus from 14 to 63 days after KA induction compared to control rats, where the PML has an obvious augment on days 28 and 63, whereas the GCL of the DG exhibits no obvious changes. *p < 0.05; **p < 0.01; ***p < 0.001; Kruskal‐Wallis with Dunn's multiple comparisons test. Data are expressed as the mean ± SEM. *n* = 18 images from six rats per group with three brain sections per rat. Scale bar = 10 μm.

Microglia are thought to inappropriately activate and prune excess synapses during the development of other neurological diseases.^53^, ^54^ To further characterize microglia‐mediated synapse pruning in TLE, IBA1, CD68, and vGluT1 or IBA1, CD68, and GAD65 triple staining was performed to determine the specificity of microglial phagocytosis (Figure 5A,B). In TLE rats 28 and 63 days after KA induction, quantification of CD68 and vGluT1 immunofluorescent signals within IBA1^+^ microglia, as well as the quantification of CD68^+^ microglia with engulfed vGluT1 revealed that microglia did not prune excitatory synapses (Figure 5C). Rather, quantification of CD68 and GAD65 immunofluorescent signals within IBA1^+^ microglia indicated a preference for pruning inhibitory synapses during KA‐induced epilepsy (Figure 5D, S3). Moreover, the quantitative analysis indicated a significant increase in the number of CD68^+^ microglia with engulfed GAD65 signal from days 14 to 63 in the CA1 and CA3 (CA1 SP: **p < 0.01, 1.33 ± 0.21 vs. 0.00 ± 0.00, 14 days vs. control group, ***p < 0.001, 2.50 ± 0.39 vs. 0.00 ± 0.00, 28 days vs. control group, ***p < 0.001, 3.06 ± 0.56 vs. 0.00 ± 0.00, 63 days vs. control group; CA3 SP: ***p < 0.001, 2.83 ± 0.58 vs. 0.17 ± 0.09, 14 days vs. control group, ***p < 0.001, 3.56 ± 0.54 vs. 0.17 ± 0.09, 28 days vs. control group, ***p < 0.001, 3.83 ± 0.43 vs. 0.17 ± 0.09, 63 days vs. control group; SR: **p < 0.01, 1.00 ± 0.18 vs. 0.06 ± 0.06, 14 days vs. control group, ***p < 0.001, 2.56 ± 0.47 vs. 0.06 ± 0.06, 28 days vs. control group, ***p < 0.001, 2.11 ± 0.42 vs. 0.06 ± 0.06, 63 days vs. control group; SL: **p < 0.01, 2.83 ± 0.44 vs. 0.17 ± 0.09, 14 days vs. control group, ***p < 0.001, 7.67 ± 0.75 vs. 0.17 ± 0.09, 28 days vs. control group, ***p < 0.001, 3.61 ± 0.56 vs. 0.17 ± 0.09, 63 days vs. control group, Figure 5E,F,H,I). In the PML of DG, plentiful engulfed microglia were detected on days 28 and 63 (***p < 0.001, 4.50 ± 0.61 vs. 0.11 ± 0.08, 28 days vs. control group, ***p < 0.001, 5.39 ± 0.61 vs. 0.11 ± 0.08, 63 days vs. control group, Figure 5G). In the GCL of DG, though, no significant change was observed after the KA induction (Figure 5J). These data supports a selective pruning of synapses by microglia during epileptogenesis.

### 3.6 Microglia selectively prune inhibitory synapses in the hippocampus in TLE human patients

**FIGURE 6:**
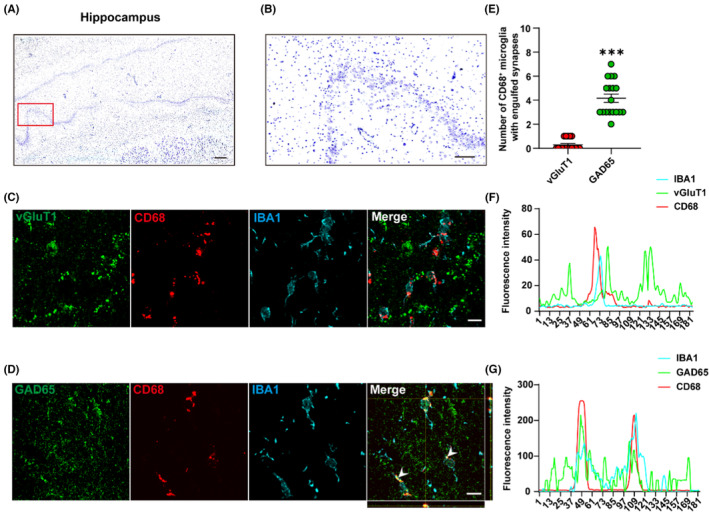
The selective pruning of inhibitory synapses by microglia is validated in the hippocampus in mTLE patients. (A, B) A representative Nissl staining of the caudal levels of DG from the hippocampus of a patient with mTLE (A: scale bar = 500 μm; B: scale bar = 100 μm). (C, D) Representative confocal images are depicted for CD68 (red), vGluT1 (green), IBA1 (cyan) and CD68 (red), GAD65 (green), and IBA1 (cyan) immunoreactive puncta in the SR of the hippocampus in TLE patients. An orthogonal view of the confocal image revealed the colocalization of IBA1 (cyan), CD68 (red), and GAD65 (green), suggesting that microglia preferentially prune inhibitory synapses in the hippocampus of mTLE patients (scale bar = 10 μm). (E) Quantification of the number of CD68^+^ microglia with engulfed GAD65 or vGluT1 signals shows a greater number of CD68^+^ microglia with engulfed GAD65 than with engulfed vGluT1 in mTLE patients. (F, G) Quantification of CD68 and vGluT1 immunofluorescent signals in IBA1^+^ microglia indicated no colocalization of lysosomes and excitatory synapses, while quantification of CD68 and GAD65 immunofluorescent signals within IBA1^+^ microglia demonstrated phagocytosis of inhibitory synapses by microglial lysosomes in mTLE patients. ***p < 0.001; Kruskal‐Wallis with Dunn's multiple comparisons test. Data are expressed as the mean ± SEM. *n* = 18 images from six patients per group with three samples per patient.

To further validate the selectivity of microglial phagocytosis in the human mTLE hippocampus, we next collected 18 brain tissues resected from six patients with drug‐resistant mTLE by performing co‐immunostaining of IBA1, CD68 and vGluT1 or IBA1, CD68 and GAD65. Representative images are presented below (Figure 6C,D). Consistent with the results observed in the rat model of KA‐induced TLE, quantification of CD68, and vGluT1 immunofluorescent signals within IBA1^+^ microglia showed no colocalization of lysosomes and excitatory synapses (Figure 6F), while quantification of CD68 and GAD65 immunofluorescent signals within IBA1^+^ microglia revealed phagocytosis of inhibitory synapses by microglial lysosomes in TLE patients (Figure 6G). Quantification of the number of CD68^+^ microglia with engulfed GAD65 or vGluT1 signals also revealed the preferential pruning of GAD65 by microglia, rather than vGluT1(***p < 0.001, 4.17 ± 1.35 vs. 0.28 ± 0.11, GAD65 vs. vGluT1, Figure 6E). Together, immunostaining in human tissue provides compelling evidence that microglia can modify the present neural circuits through specific phagocytosis of inhibitory synapses in TLE.

## 4 DISCUSSION

In this study, we conducted detailed research to determine how the number of excitatory and inhibitory synapses changed at multiple typical time points following KA induction. We discovered that the total area of vGluT1 in circuit‐related subregions of the hippocampus, especially in the SR, SL and PML, presented a continuous increase during epileptogenesis, reflecting the general rise in excitability in the epileptic hippocampus. The total area of GAD65 in SL and PML, however, showed a remarkable downward trend, which implies a decline in inhibitory activity. Furthermore, microglia were activated in a considerable number after the formation of SRSs, most of which conduct active synaptic phagocytosis. Although the number of activated microglia decreased 63 days after KA induction, when rats typically develop HS, there were still plentiful microglia exhibiting phagocytosis function. Moreover, we found that microglia preferentially pruned inhibitory synapses after the formation of SRSs, which contributed to the alteration of synaptic numbers. Finally, we validated the selective pruning of inhibitory synapses by microglia in human hippocampal tissues.

Glutamate is a prominent neurotransmitter in the brain that mediates excitatory synaptic transmission and is essential for synaptic plasticity.^55^ We used vGluT1 to label excitatory synapses to investigate the variation in the number of excitatory synapses. We discovered that the number of excitatory synapses in the PML of the DG increased dramatically from days 7 to 28 after KA induction, which may indicate an increase in excitatory projections from the PML to the SL during epileptogenesis. In the DG of seizure‐sensitive gerbils, vGluT1 immunoreactivity was also enhanced. This is consistent with mossy fiber sprouting (MFS), a typical pathological change in epilepsy. Following epileptic damage by KA injection, mossy fibers lose projections from pyramidal cells in the CA3 and hilus and thus sprout abnormally into the IML and SL to reconstruct the synapse network.^56^, ^57^ This increase in excitatory input peaked on day 28, which is consistent with the sprouting number of mossy fibers, as previous studies in a model of KA‐induced epilepsy in rats found that the density of abnormal synaptic connections formed by MFS was significantly lower at 7 days after status epilepticus than at 28 days.^58^ The number of excitatory synapses decreased to the control level on day 63, which may be attributed to the massive neuronal death associated with HS 63 days after KA injection in rats. However, there was no significant change in the number of excitatory synapses in the GCL at any time point. Similar results were also found in a pilocarpine‐induced rat model, where there was a tiny difference in granule cell activity between pilocarpine‐induced and control rats.^57^ This may be interpreted as the massive death of neurons in the hilus after epilepsy, causing the loss of projection targets in the GCL.^59^ However, specific mechanisms still need to be further studied. Dendrites of pyramidal cells in the CA3 receive excitatory projections from mossy fibers, resulting in a significant rise in the number of excitatory synapses in the SP of CA3, as well as in the SL from days 7 to 28. The number of synapses at 63 days fell for the same reason stated above. The pyramidal cells in the CA3 then made synaptic contact with the dendrites of CA1 pyramidal cells through Schaffer collateral, and the number of excitatory synapses in the SP and SR of the CA1 increased accordingly. Hence, our study correlates changes in the number of excitatory synapses with synapse circuits in the hippocampus, providing a new idea on circuit‐based interventions to treat epilepsy.

Two main types of neurons exist in the hippocampus: glutamatergic pyramidal neurons and GABAergic inhibitory interneurons.^22^ With immunostaining in a rat epilepsy model and human samples, we found that microglia preferentially pruned inhibitory synapses. This may explain why the number of inhibitory synapses in the SL and PML declined significantly from days 7 to 63 after KA induction. Previous studies also found that the onset of seizures reduced gephyrin expression in the hippocampus and adjacent neocortex.^24^, ^60^ However, the number of inhibitory synapses in the SP of the CA3 rose significantly from days 7 to 28. Our study focused on the changes in synapses and the selective phagocytosis of microglia. However, they may not have the necessary and sufficient relationships. Prior research found that GAD65 expression in the CA1 and CA3 was significantly increased by 4 weeks in a rat model of PTZ‐induced epilepsy.^61^ This compensatory increasement suggested a self‐protection mechanism in epilepsy.^62^ Based on this, it is presumably because the drop in inhibitory synapses in the SL reduced the inhibitory projections received by CA3 pyramidal neurons. To restore the E/I balance, the brain thus upregulated the inhibitory synaptic regeneration in the SP to reconstruct synaptic connections. In addition, we observed a large increase in the number of inhibitory synapses in the SP and SR of CA1 14 and 28 days after KA induction. As a result of the increase of inhibitory synapses in the SP of the CA3, the inhibitory projection via Schaffer's veins may correspondingly increase.^63^ Furthermore, the phagocytosis of microglia also differed in different regions and at different time points. Studies found that the hippocampal seizure core migrated slowly in epileptic rats,^12^ which suggested that the progression of epilepsy may lead to the propagation of the firing regions in the trisynaptic circuit. Our data did not show a significant increase of phagocytic microglia in the SP and SR of the CA1 until 14 days after KA induction. Although there was a higher quantity of activated microglia on day 14, the number of CD68^+^ microglia was considerably less than that on days 28 and 63. On this ground, it is possible that day 28 was the time point when microglia perceived the abnormal discharge of the CA1 and began to conduct massive synaptic pruning. Studies have found that microglia conduct massive phagocytosis in patients with mTLE.^64^ Our studies also demonstrate the synaptic pruning of microglia in rats 63 days following KA treatment.^34^ Thus, the compensatory increase of inhibitory synapses in the SP and SR of CA1 could be predominant, which may result in the temporary increase of inhibitory synapses before day 63. Intriguingly, according to electrophysiological investigations, animal models of epilepsy exhibited reduced inhibition of CA1 pyramidal cells.^65^ Given the inhibitory presynaptic marker that we employed, it is also necessary to incorporate postsynaptic markers to examine the number of functional synapses.

Microglia regulate the equilibrium of synapses during neurodevelopment by creating new synapses and removing non‐functional ones.^66^ According to our findings, the number of microglia was highly activated during spontaneous seizures. This is consistent with earlier research indicating that during the chronic phase of epilepsy, microglia were strongly active.^67^ However, activated microglia might not possess phagocytic function; therefore, we co‐stained microglial markers with lysosomal markers to quantify the number of phagocytic microglia. Interestingly, 14 days post‐KA induction, the number of activated microglia exhibited a great quantity, but the number of CD68^+^ microglia was well below that on days 28 and 63, which reveals that microglia began to conduct prominent phagocytosis on day 28.^34^, ^64^ In addition, 63 days post‐KA induction, the number of activated microglia reduced significantly compared to 28 days, which may be due to a large amount of neuronal death and synaptic damage that occurred during HS, which led to a considerable decrease in the number of microglia. A prior study also supports this phenomenon, which found amoeboid microglia expressed at a low level in human epileptic temporal lobes.^39^ In addition, the weakened activation of microglia was accompanied with neuron loss in a mouse model of TLE.^68^ However, CD68^+^ microglia still surpassed the control and were close to that on day 28, suggesting that microglia continued to conduct phagocytosis even during HS, which consisted of a major cause of cognitive impairment in epilepsy. Notably, when comparing the quantity of microglia in the CA1 with that in the CA3 and DG 28 days after KA induction, we discovered that the numbers of activated microglia and CD68^+^ microglia were substantially higher in the CA3 and DG than in the CA1. This is in line with the substantial neuronal death found in CA3 and DG in rats treated with KA, indicating the significance of CA3 and DG in the development of epilepsy.^69^ In addition, we found that microglia preferentially pruned inhibitory synapses in epileptic brains. It is noteworthy that although the number of activated microglia on day 14 was considerably more than that on day 63, CD68^+^ microglia or microglia with engulfed GAD65 signal on days 28 and 63 were superior to that on day 14. Considering the statistical analysis above, it can be ratiocinated that microglia did not exert noticeable phagocytosis until 28 days after the KA induction. We also utilized human samples to demonstrate the validity of this conclusion. However, the mechanism of the preferential pruning by microglia is still obscure. Studies indicate that the synaptic pruning of microglia could be regulated by the classical complement molecules C3 and C1q.^64^ Microglia preferentially prune inhibitory synapses in progranulin‐deficient mice by activating C1q.^70^ C1q was abundantly deposited and co‐localized with inhibitory synapses engulfed by microglia in the multiple sclerosis (MS) hippocampus.^71^ In addition to disease models, GABA‐receptive microglia also remodel C1q tagged inhibitory synapses in mouse postnatal development.^72^ Our prior research also found that intranasal infusion of C3a reversed microglia‐mediated synaptic phagocytosis in epilepsy after captopril treatment.^34^ These results suggest that microglia during SRS may highly express GABA receptors and C1q may act as an “usher” to help microglia recognize inhibitory synapses, all of which deserve further investigation.

We applied presynaptic markers (vGluT1 and GAD65) to investigate changes in the number of excitatory and inhibitory synapses. Notwithstanding, combining presynaptic markers with postsynaptic markers can better reflect the function of synapses in neural circuits,^73^ which will be further explored. In addition, electrophysiological studies may be added to confirm changes in the number of synapses in the future. In fact, postsynaptic density‐95 protein (PSD95) exhibited decreased expression in epileptic rats.^74^ Additionally, we noticed that some Iba^+^ puncta colocalized only with synaptic puncta but not with CD68. Actually, in addition to phagocytosis, microglia generate microglial process pouches that enveloped the dendrites of neurons in mice treated with KA for 6 h without performing phagocytosis.^75^ In complex febrile seizures, microglia could also displace GABAergic synapses by P2Y_12_ receptor.^66^ Thus, the function of microglia on synapses in epilepsy may be bidirectional. The specific role may be carried out depending on the stage of disease progression. Moreover, our study only tested male SD rats. However, the reactivity of microglia can differ in sex dimorphism, given microglia adopted ameboid morphology and exhibited increased phagocytic capacity only in the developing brains of males, while females possess decreased phagocytic capacity.^76^ Furthermore, cerebrovascular reactivity reproducibility was slightly higher in males.^77^ And there was a higher expression level of proteins involved in neurovascular metabolism and oxidative respiration in females, which is an essential element for the phagocytic function of microglia.^78^, ^79^ Therefore, studying both sexes should be a future subject.

In sum, our study demonstrated that microglia selectively pruned inhibitory synapses to modify neuronal circuits in the hippocampus, resulting in changes in the number of excitatory and inhibitory synapses, which ultimately led to E/I imbalance and epilepsy formation. Our findings highlight a biological mechanism by which microglia contribute to synapse degradation. This underscores the importance of microglia in the pathophysiology of epilepsy and implies that microglia can be targeted for the prevention and treatment of epilepsy in clinical settings.

## AUTHOR CONTRIBUTIONS

JF, XD, LZ, YX, and XH conceived and designed the study. JF, XD, and DL performed the experiments and analyzed the results. JF and YX wrote and revised the manuscript. XW, JJ, LG, CC, AX, and WS participated in the data acquisition, analysis, and interpretation. YX and LZ provided funding support.

## FUNDING INFORMATION

This work was supported by Grants from Key R&D Program Project of Zhejiang, Grant/Award Number: 2022C03034, the National Natural Science Foundation of China (82001203 to YX), Scientific Research Foundation of Zhejiang University City College, Grant/Award Number: X‐202103.

## CONFLICT OF INTEREST STATEMENT

The authors have no financial conflicts of interest.

## CONSENT FOR PUBLICATION

Not applicable.

## Supporting information


Fig. S1.

## Data Availability

The datasets used and/or analyzed during the current study are available from the corresponding author on reasonable request.
