# Supplementary material for: Preferential pruning of inhibitory synapses by microglia contributes to alteration of the balance between excitatory and inhibitory synapses in the hippocampus in temporal lobe epilepsy
Source: CNS Neurosci Ther. 2023 Apr 18;29(10):2884–900. doi: 10.1111/cns.14224 (PMC10493672; doi:10.1111/cns.14224)
Supplement: Supplementary file 1 — Fig. S1. [file CNS-29-2884-s001.docx]

**
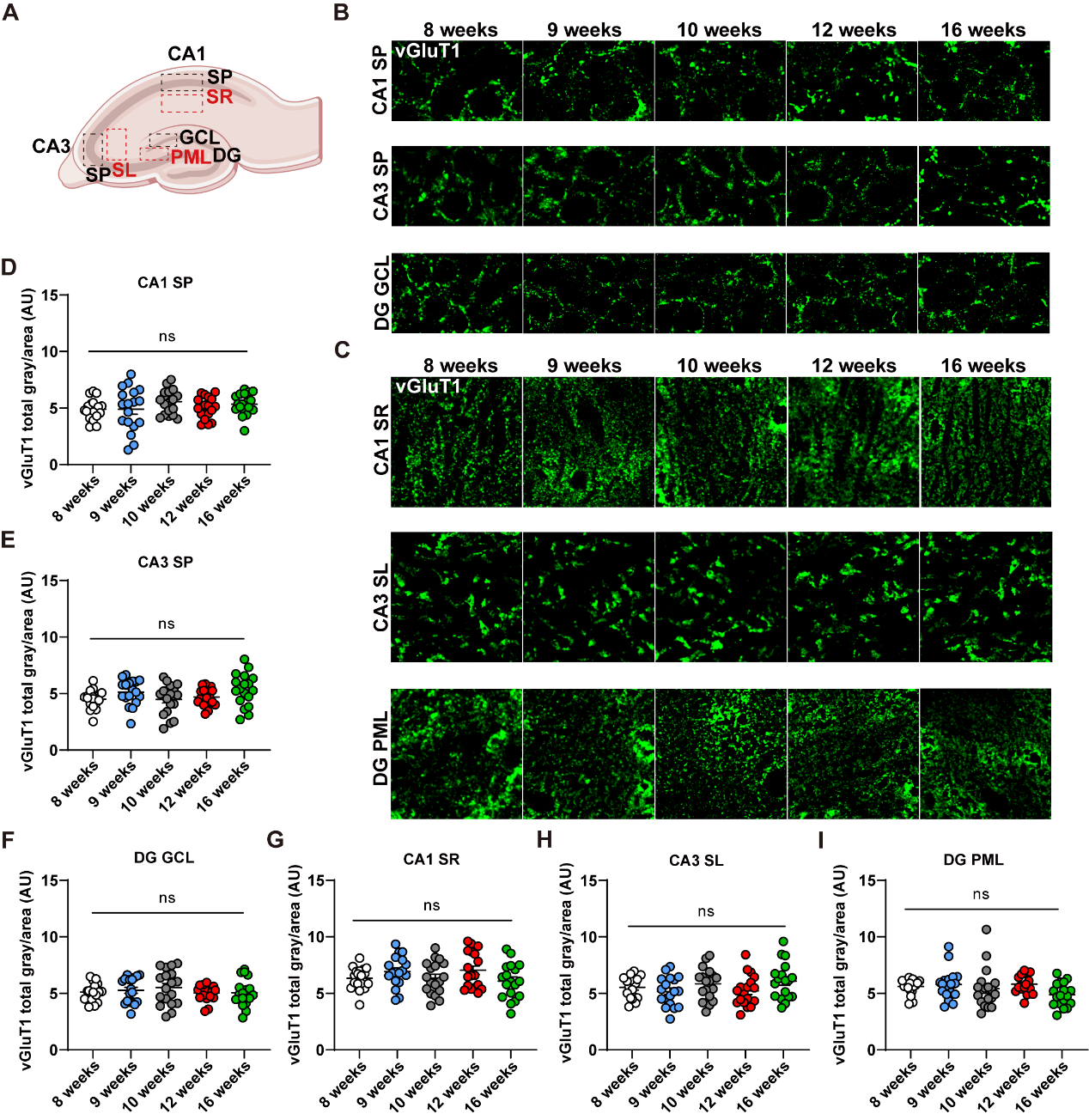
Fig. S1. The stable amount of vGluT1 in control rats’ hippocampus at the age from 8 to 16 weeks.** (A) The regions of interest are shown in the hippocampus in the schematic diagram. (B, C) Representative vGluT1 (green) immunostaining from the functional regions of the CA1, CA3 and DG in age-matched control. Scale bar = 10 μm. (D-I) Quantification of the total area of vGluT1 reveals no obvious changes at various ages. **p* < 0.05, ***p* < 0.01, ****p* < 0.001, one-way ANOVA with Tukey’s post hoc test. Data are expressed as the mean ± SEM. N = 18 images from 6 rats per group with 3 brain sections per rat.

**
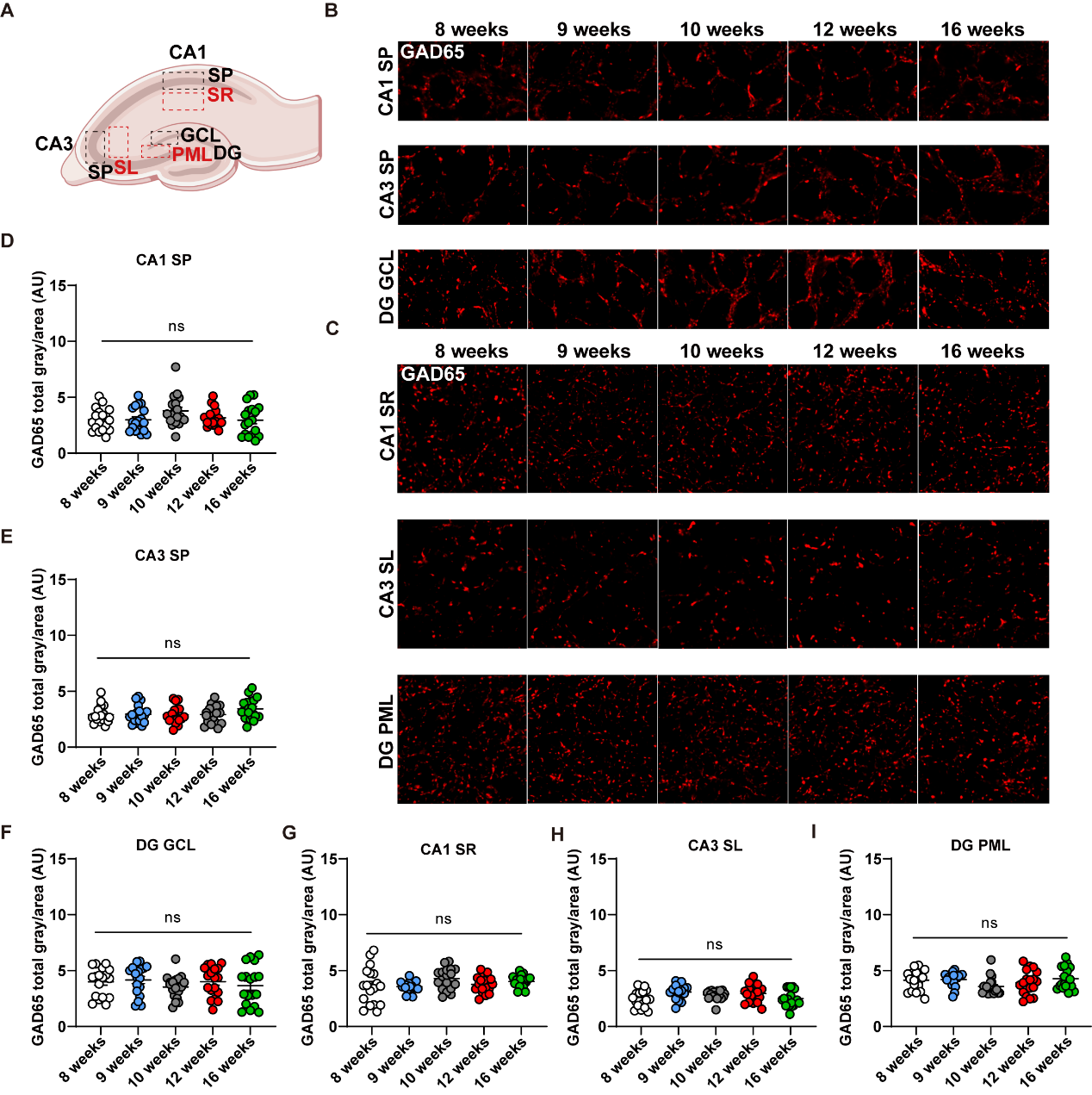
Fig. S2. The stable amount of GAD65 in control rats’ hippocampus at the age from 8 to 16 weeks.** (A) The regions of interest are shown in the hippocampus in the schematic diagram. (B, C) Representative GAD65 (red) immunostaining from the functional regions of the CA1, CA3 and DG in age-matched control. Scale bar = 10 μm. (D-I) Quantification of the total area of GAD65 reveals no obvious changes at various ages. **p* < 0.05, ***p* < 0.01, ****p* < 0.001, one-way ANOVA with Tukey’s post hoc test. Data are expressed as the mean ± SEM. N = 18 images from 6 rats per group with 3 brain sections per rat.

**
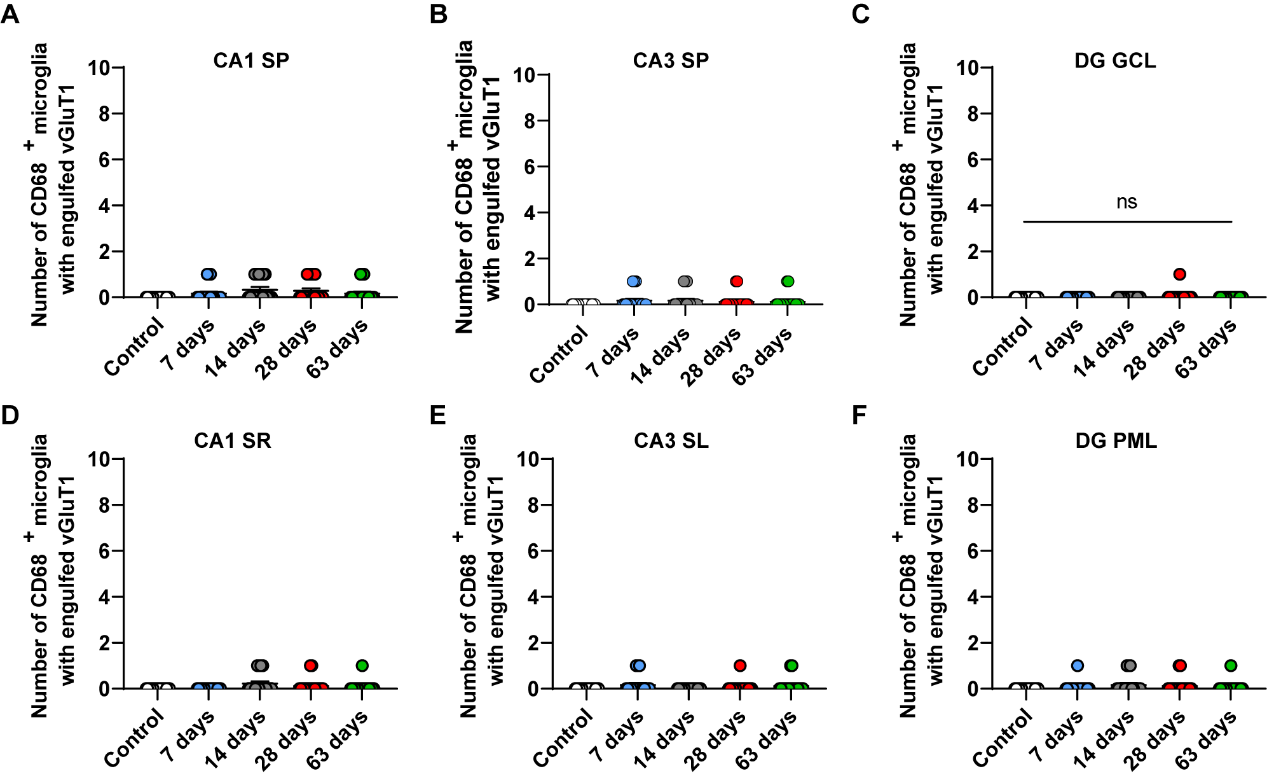
Fig. S3. Kainic acid-induced activated microglia prunes scanty excitatory synapses in the rats’ hippocampus.**

(A-F) Quantification of the number of CD68+ microglia with engulfed vGluT1 presents no obvious changes across several time points after the KA induction. *p < 0.05; **p < 0.01; ***p < 0.001; Kruskal-Wallis with Dunn's multiple comparisons test. Data are expressed as the mean ± SEM. N = 18 images from 6 rats per group with 3 brain sections per rat.
